# A Two-Phased Pilot Study Evaluating the Feasibility and Acceptability of the Cognitively Enriched Walking Program “Take a Walk with Your Brain” for Older Adults

**DOI:** 10.1155/2024/2438067

**Published:** 2024-04-10

**Authors:** Pauline Hotterbeex, Melanie Beeckman, Pieter-Jan Marent, Julie Latomme, Fien De Block, Lauren De Baets, Sebastien Chastin, Greet Cardon, Jannique G. Z. van Uffelen

**Affiliations:** ^1^Department of Movement Sciences, Leuven Brain Institute, KU Leuven, Leuven, Belgium; ^2^Department of Movement and Sports Sciences, Ghent University Research for Aging Young, Ghent University, Ghent, Belgium; ^3^Artevelde University of Applied Sciences, Ghent, Belgium; ^4^School of Health and Life Sciences, Glasgow Caledonian University, Glasgow, UK

## Abstract

Given the aging population, finding solutions to retain optimal cognitive capacity is a research priority. The potential of physical activity to reduce the risk of cognitive decline and to enhance cognitive functioning is established. Combining physical with cognitive activity has been put forward as a potentially even more effective way to promote healthy cognitive aging. Most studies on combined interventions have however been conducted in laboratory settings. This paper reports on a two-phased pilot study evaluating the acceptability and feasibility of a newly developed real-life cognitively enriched walking program for adults aged 65+ years. In Phase I, the feasibility and enjoyability of the cognitive tasks was evaluated by conducting walk-along interviews with older adults (*n* = 163). In Phase II, the cognitively enriched walking program was piloted in two groups of older adults (*n* = 19), and the feasibility and acceptability of the program and cognitive tasks was evaluated by means of questionnaires and focus groups. The cognitive tasks (i.e., median scores of ≥3 on a total of 4 (Phase I) and ≥6 on a total of 10 (Phase II) for most of the tasks) and the cognitively enriched walking program (i.e., median scores of ≥7 on a total of 10) were considered feasible and acceptable. Based on the input of the participants, key considerations for a feasible and acceptable program were defined: participants should be sufficiently challenged cognitively and physically, social interaction is an important motivator, cognitive tasks should make use of stimuli reflecting daily life and be conducted in group, the rationale for the tasks should be explained to participants, the frequency of the group sessions should be maximum 2 times a week, and the program should be supervised by a trained coach. These results warrant future research to establish the effectiveness of this program.

## 1. Introduction

As a result of declined fertility rates and increased life expectancy, the proportion and actual number of older adults has increased considerably over the past decades, and this increase is projected to continue. In 2021, those aged 65 years or older accounted for 20.8% of the population of the European Union, while this proportion is expected to rise to 31.3% by 2100 [1]. Life expectancy is also rising and has increased in Europe from 77.6 years in 2002 to 80.4 years in 2020 [2]. However, these additional life years are not necessarily lived in good health. In Europe, healthy life expectancy (i.e., the number of years that a person is expected to continue to live in a healthy condition) was 64.5 years for women and 63.5 years for men in 2020 [3].

Healthy aging, defined by the World Health Organization (WHO) as “the process of developing and maintaining the functional ability that enables wellbeing in older age,” is therefore considered a key challenge of this decade. Retaining optimal cognitive capacity (i.e., a person's capacity to perform a range of mental functions such as memory and executive functions) is a critical aspect contributing to healthy aging [4, 5]. Globally, the number of older adults living with dementia was estimated to be 55 million in 2019 and is anticipated to rise to 139 million by 2050, which underpins the importance of cognitive health today and in the future [6]. Thus, investigating ways to retain optimal cognitive health at older age and consequently preserving older adults' quality of life is more important than ever.

Although age-associated nonpathological cognitive decline is inevitable, there is an individual variability in the extent and rate of cognitive decline [7–9]. Maintaining optimal cognitive health is partly determined by unmodifiable factors such as genetics, but research has also identified several modifiable “lifestyle” risk factors, such as engagement in physical activity (PA) and cognitive activity (CA) [10–13]. These factors can positively impact cognitive health at older age and are found to decrease the risk to develop dementia [10–12].

Several systematic reviews and meta-analyses have pointed towards positive association and effects of both physical activity and exercise for cognitive function. For example, a recent systematic review and meta-analysis of 58 observational studies showed that higher PA levels are associated with a 20% decrease in the risk of dementia (pooled relative risk = 0.80; 95% CI [0.77–0.84]) when compared with a reference or inactive group [14]. Of all exercise (i.e., a structured form of PA) types, the effects of aerobic exercise and resistance training on cognitive functioning have been studied most extensively and have been found to positively affect cognitive functioning (i.e., executive functioning, memory, and attention) in older adults [15, 16]. Furthermore, an umbrella review by Erickson et al. [15] has shown a moderate effect of long-term moderate-to-vigorous PA interventions (i.e., more than one session) on cognitive outcomes in adults aged 50 and older (i.e., Hedges' *g* ranging from nonsignificant to 0.48). There are several potential pathways for the beneficial effect of PA on cognitive function. Physiologically, these effects could be attributed to the PA-induced neuroplasticity, through an increase in Brain-Derived Neurotrophic Factor and Insulin-Like Growth Factor which results in positive changes in the brain, such as the promotion of neurogenesis, synaptogenesis and angiogenesis, and improved brain structure and functional connectivity [17].

Recently, it has been hypothesised that an even more effective strategy to preserve cognitive functioning in older adults is to combine PA (e.g., aerobic treadmill walking) with CA (e.g., (computerized) cognitive training). Examples of combined physical and cognitive activity (PA + CA) are doing memory exercises while walking on a treadmill [18], exergaming [19], or sequentially combining physical training and cognitive training [20–22]. Indeed, three meta-analyses concluded that larger cognitive gains can be achieved with cognitively enriched PA intervention programs, compared to PA programs without cognitive enrichment (i.e., Hedges' *g* in the three meta-analyses ranging from 0.15 to 0.24) [23–25]. Although most studies found no additional effect on cognitive functioning of PA + CA in comparison with CA alone [23–25], PA + CA also fosters benefits for physical function (i.e., functional mobility), which is not the case for CA alone [23]. Physiologically, it is assumed that PA and CA have a synergistic impact on neuroplasticity, in which PA facilitates neuroplasticity (e.g., through neurogenesis and synaptogenesis) and CA guides the neuroplasticity by promoting the survival and integration of newly formed neurons [26–30].

A major shortcoming of current research on PA + CA interventions is, however, that most studies have been conducted in controlled laboratory settings [23–25]. To combat the increasing rate of cognitive decline and dementia in the aging population, real-life (i.e., in a more natural, everyday setting, as opposed to in controlled laboratory settings), low-cost, and scalable PA + CA programs that are easily accessible to the majority of the older population are needed. Recent research focused on home-based PA + CA programs [21, 22, 31]. These home-based programs are either complete individual programs [21] or combine a number of group and (home-based) individual sessions [22]. Nevertheless, Zhu et al. [25] found larger effects for group-based PA + CA interventions in comparison with individual PA + CA interventions. Furthermore, social engagement has also been shown to be linked with a lower risk of dementia [11, 32]. Additionally, it has been suggested that performing the cognitive and physical activity simultaneously might be important for the synergistic effects of PA + CA interventions [27]. Indeed, the previously mentioned meta-analyses all found simultaneous training to be more beneficial for cognitive functions compared with sequential training [24, 25] or exergaming [23].

Therefore, a real-life, group-based, simultaneous PA + CA intervention, more specifically a cognitively enriched walking program for older adults (i.e., aged 65 years and above), “Take a walk with your brain”, was recently codesigned using two complementary methodologies: (1) a multistage Delphi study with academic experts in the field of cognition, physical activity, and aging and (2) a survey study in older adults and walking coaches. This entire conceptualization process has been described in our earlier publication [33]. This newly developed cognitively enriched walking program consists of supervised group-based cognitively enriched walking sessions (i.e., simultaneously walking and performing cognitive tasks).

The current paper reports on the acceptability and feasibility of the “Take a walk with your brain” program in community-dwelling older adults aged 65+ years. This study was two-phased, focusing on the evaluation of performing cognitive tasks while walking (Phase I and Phase II), and of the cognitively enriched walking program as a whole (Phase II). Specifically, it was of our interest to know how the participants perceived the cognitive tasks and the program, and what aspects of the program were perceived as the most important to them. The research questions were as follows: (1) Do older adults perceive the cognitive tasks as feasible and acceptable? (2) Do older adults consider the whole program feasible and acceptable? and (3) What are the most important factors that contribute to a feasible and acceptable real-life cognitively enriched walking program in group?

## 2. Methods

### 2.1. Study Design

We conducted this acceptability and feasibility study in two phases. Phase I was a preparatory phase during which walk-along interviews (i.e., questions posed while walking) with one or two older adults at a time were conducted to evaluate the feasibility and acceptability of the cognitive tasks conceptualized by Marent et al. [33]. In Phase II, the *group-based* cognitively enriched walking program was piloted to evaluate the feasibility and acceptability of (i) the cognitive tasks (performed in group as opposed to alone or in pairs in Phase I) and (ii) the group program in general (i.e., a more general evaluation of performing cognitive tasks in group while walking, supervised by a coach). To obtain a comprehensive evaluation of the cognitive tasks and the group-based cognitively enriched walking program, a *mixed*-*method embedded study design* was used [34]. This design typically complements or elaborates on quantitative data with qualitative data. In the present study, rating scales were used to evaluate the cognitive tasks (Phases I and II) and the cognitive walking sessions (Phase II) (quantitative). Open-ended questions in questionnaires (Phases I and II) and focus groups (Phase II) were used to obtain deeper insight in the underlying reasons of participants' ratings and their experiences (qualitative).

Conducting the present study in two phases was an unforeseen amendment to meet COVID-19 regulations. Originally, this study was planned to take place in the summer of 2020. To meet the COVID-19 restrictions, the design was changed from an intended pilot testing in group, to a two-phased design. Phase I consisted of walk-along interviews in the fall of 2020, as these met the COVID-19 restrictions in force at that time. Phase II was a group-based pilot testing conducted in the fall of 2021, after the COVID-19 restrictions were lifted.

### 2.2. Participants

The inclusion criteria for participation in this study were being (1) 65 years or older, (2) Dutch-speaking, (3) able to walk for approximately one hour, and (4) reporting no severe cognitive, mental, or physical disorders. Recruitment was done separately for both phases by means of convenience and snowball sampling. As a part of an educational assignment, bachelor students Physical Education and Movement Sciences at two universities each recruited two older adults within their personal environment for the walk-along interviews in the period of November-December 2020 (Phase I). For the group-based program (Phase II), participants were recruited in November 2021 through the distribution of an (online) information leaflet via organizations for older adults and an existing database of older adults interested in participation in studies of our research groups. In both phases, the aim was to recruit a diverse study sample with participants of different age, gender, and sociodemographic background. All participants provided written informed consent. This study was approved by the Ethical Committee Research UZ/KU Leuven (S63305) and the Ethical Committee of Ghent University Hospital (2019/1045 BC-5773) and was carried out according to the guidelines for Good Clinical Practice (ICH/GCP) and the Declaration of Helsinki. Data collection took place between November and December 2020 for Phase I and between November and December 2021 (questionnaires) and January and February 2022 (focus groups) for Phase II.

### 2.3. Procedure

#### 2.3.1. Phase I

During each walk-along interview, older adults (i.e., alone or in pairs) walked for approximately 60 minutes with an interviewer and performed two or three cognitive tasks. The cognitive tasks were randomly assigned across participants. An overview of the cognitive tasks can be found in Table 1 (for detailed descriptions, see the article of Marent et al. [33]). A standardized protocol for each cognitive task was prepared for the interviewers. It included a description of the content, instructions, materials, required preparations, and general attention points based on input from the developmental stage of this project [33]. Every walk-along session started with 10–15 minutes of brisk walking, after which the instructions for the first cognitive task were communicated, and the participant(s) performed the cognitive task. After performing the first task, participants were questioned about how they experienced performing the cognitive tasks by means of a structured walk-along interview consisting of rating scales and open questions (for more information, see infra, *Quantitative program evaluation measures*). The same process was repeated for the second and, if applicable, third cognitive task. At the end of the walk-along interview, there was some time for relaxed walking (i.e., a cooling-down). In total, about 15–20 minutes per 30 minutes of the walk were spent on the performance of the cognitive tasks. Participants' responses to all questions and general sociodemographic information of the participant were registered by a researcher on a standardized response form.

#### 2.3.2. Phase II

Before starting the group-based cognitively enriched walking program, participants were asked to complete an online questionnaire to collect sociodemographic information, general health-related information, information on their level of physical activity, psychosocial health, and their subjective perception of their cognitive functioning (see infra, *Sociodemographic and general health-related measures*). A cognitive test battery and accelerometery were used in a subsample to confirm their feasibility for use in the future evaluation of the effectiveness of this program in a randomized controlled trial. As this goes beyond the scope of this paper, we will not report on this.

Participants in Phase II took part in six sessions of the cognitively enriched walking intervention in a group of approximately ten older adults. There were two walking groups: one in the city of Ghent (*n* = 10) and the other in the city of Leuven (*n* = 9). The sessions were organized with a frequency of twice a week over a total time period of three weeks, in November-December 2021. They were supervised by certified walking coaches who completed a formal training of “Wandelsport Vlaanderen” (Walking Federation Flanders), meaning they had already developed the necessary competences to guide walks before participating in this study. The two groups had different coaches, but the same coach supervised all six sessions for each group. After each of the six walking sessions, participants were asked to complete a program evaluation questionnaire consisting of rating scales and open-ended questions (see infra, *Quantitative program evaluation measures*). The attendance was registered by a researcher (who was present during all sessions). After the six sessions, focus groups were organized separately for participants of both walking groups in order to gain an in-depth evaluation of the cognitively enriched walking program. By combining questionnaires and focus groups, potential bias caused by social desirability effects was reduced.

As this was a real-life program, the settings differed from a recreational park to more urban settings. Each session lasted approximately 60 minutes and, as conceptualized by Marent et al. [33], consisted of three parts: (1) a warm-up of 5–10 minutes (brisk) walking, (2) about 15–20 minutes of cognitive tasks per 30 minutes of walking (i.e., two to three cognitive tasks), and (3) 5–10 minutes relaxed walking. As it was not the aim to compare evaluations of the cognitive tasks between the two groups, it was most efficient to let them be evaluated by only one group. Therefore, the cognitive tasks were divided into 11 combinations of two or three cognitive tasks in a way to stimulate different cognitive functions within each session. Both groups had the same introductory session with the aim to start off the program and getting to know each other while performing cognitive tasks. For instance, participants had to remember facts about each other in the cognitive task “Facts and titbits.” The remaining five sessions were different for the two groups. An overview of the combinations of tasks can be found in Table 2, as well as information on which group evaluated which session.

Before the start of the sessions, the coaches received a detailed description of all cognitive tasks including an explanation of the task, instructions, duration of the task, possible variations, materials needed, preparations needed, how to guide the task, and important considerations (for an example, see Supplementary File 1). First, they received this digitally, so they could read this in preparation of a face-to-face meeting with the researchers during which all cognitive tasks, how to combine these tasks with walking, and any questions and concerns the coaches had were discussed. The face-to-face meeting was organized separately for the coaches of the two groups and lasted approximately 4 hours. After this meeting, the coaches received a printed version of this manual. To use as a prompt during the walking sessions, the coaches also received summary cards which consisted of a checklist and the different parts of the walk (e.g., first approx. 10 minutes brisk walking, then cognitive task 1, cognitive task 2, and at the end approx. 10 minutes relaxed walking; for an example, see Supplementary File 2). During each session, the coach explained and guided the correct execution of the cognitive tasks. One of the researchers was present during all sessions to ensure correct adherence to the intervention protocol. This served as a form of fidelity check for the implementation of the intervention. If needed, the coach and researchers had a short meeting before and/or after each session, in which potential difficulties or questions that came up during the preparation or execution of the session could be discussed. If necessary, small changes were made accordingly (e.g., a cognitive task was prepared to be more difficult than planned based on feedback of the participants, or a task was changed from a written to a verbal task because of practical considerations).

### 2.4. Sociodemographic and General Health-Related Measures

These data were gathered for descriptive purposes only. All participants (Phases I and II) were asked to provide their date of birth, gender, current marital status, and highest educational degree. Phase II participants were additionally asked to provide their country of birth and current professional activity and also completed a baseline questionnaire consisting of several validated questionnaires which are explained in more detail below.

For the measurement of PA (Phase II), the Dutch version of the *International Physical Activity Questionnaire Short Form* (IPAQ-SF) [35] was used. This seven-item questionnaire with open-ended questions is one of the most used questionnaires to evaluate PA. The IPAQ-SF was evaluated to have appropriate content validity and reliability in systematic reviews of van Poppel et al. [36] and Silsbury et al. [37], respectively. Furthermore, it is a brief and low-cost measure, making it an accessible tool to estimate the PA levels of participants. Categorical scores were calculated following the Guidelines for the Data Processing and Analysis of the International Physical Activity Questionnaire [38].

Subjective cognitive functioning (Phase II) was assessed using the Dutch version of the *Cognitive Failure Questionnaire* (CFQ), which has 25 questions (e.g. “Do you have trouble making up your mind?”) on a five-point Likert scale going from “Very often (4)” to “Never (0).” The CFQ is the most widely used questionnaire to measure subjective cognitive failures [39] and has been shown to be a reliable measure [40]. It is a measure of psychological distress related to cognitive difficulties, rather than a valid measure of objective cognitive deficits. Subscales can be derived, but according to recent research recommending to only use the total score because the CFQ likely represents a single underlying construct [40], in this study the total score was used. A higher total CFQ score indicates more frequent cognitive errors reported by the participant [41–43].

Depression, anxiety, and social isolation (Phase II) were assessed through three Dutch Patient-Reported Outcomes Measurement Information System (PROMIS®) four-item short forms: Scale V1.0 Depression, Scale V1.0 Anxiety, and Scale V2.0 Social Isolation [44–46]. The response options for these four-item short forms ranged from “Never (1)” to “Always (5).” Sleep was also assessed using a PROMIS four-item short form: V1.0 Sleep Disturbances, with response options ranging either from “Very good (1)” to “Very poor (5)” or “Not at all (5 or 1)” to “Very much (1 or 5)” [46, 47]. The PROMIS short forms were scored making use of the HealthMeasures scoring service; this program calculates T-scores based on Response Pattern Scoring.

### 2.5. Quantitative Program Evaluation Measures

#### 2.5.1. Phase I

In Phase I, participants were asked to rate each cognitive task in terms of *feasibility* (i.e., “I feel capable of performing this task correctly”) and *enjoyment* (i.e., “I enjoy this task”) making use of five-point rating scales (from “Totally Disagree (0)” to “Totally Agree (4),” middle point: “Neither Agree, Nor Disagree (2)”). This information was registered by a researcher on a standardized response form.

#### 2.5.2. Phase II

In Phase II, participants evaluated both the cognitively enriched walking sessions and the cognitive tasks by means of a questionnaire after each cognitively enriched walking session. To gain a more nuanced impression of participants' experience, participants were instructed to rate every *cognitively enriched walking session in general* (i.e., “How would you rate the overall session?”), on an 11-point response scale (i.e., “Not good at all (0)” to “Very good (10),” no middle point was defined). Furthermore, the cognitive tasks were evaluated on 11 rating scales, namely, *feasibility, enjoyability, difficulty, challenge, competition* (i.e., during the cognitive task), *meaningfulness* (i.e., of the cognitive task), *interaction* (i.e., with other participants, during the cognitive task), *appropriateness for the combination with walking, appropriateness for the age group, perceived positive influence on the brain*, and* clarity of instructions* (i.e., given by the coach). An 11-point response scale going from “Totally Disagree (0)” to “Totally Agree (10)” was used (the middle point of the response scale was not defined). In these evaluation questionnaires, the cognitive tasks (1) “Buzz it” and “Problem solving” as well as (2) “Remember the route” and “Quest with environmental cues” were performed at the same time and therefore evaluated as one task.

Furthermore, attendance was registered by the researcher present during all sessions.

### 2.6. Qualitative Program Evaluation Measures

#### 2.6.1. Phase I

During the walk-along interviews, participants were asked to verbally explain *what could be done to make the cognitive task more enjoyable or feasible*. Responses of the participants were registered by a researcher on the same response form as for the quantitative evaluation of the cognitive tasks.

#### 2.6.2. Phase II

*(1) Open-Ended Questions*. In addition to the quantitative evaluation of the cognitively enriched walking session and cognitive tasks, three open-ended questions were completed after each session (i.e., “What did you like most about today's session?,” “What did you like least about today's session?,” and “How could the session be improved?”).

*(2) Focus Groups*. To gain a deeper understanding of the experiences of the participants of Phase II regarding the feasibility and acceptability of the cognitively enriched walking program, focus groups were conducted separately for each walking group. The focus groups took place in January and February 2022 and were each moderated by two researchers. A semistructured interview guide was developed for the focus groups. Both focus groups took between 90 and 120 minutes and were audio recorded. Before starting the recording, participants agreed to the focus group being recorded.

### 2.7. Data Analysis

#### 2.7.1. Quantitative

Quantitative data analyses were carried out using the Statistical Program Package for Social Sciences (SPSS) (version 28, IBM, Chicago, IL). To describe the samples, appropriate descriptive statistics were used. T-scores were calculated for the PROMIS four-item short forms as indicated above. These PROMIS T-scores were compared with the US reference values for V2.0 Social Isolation (i.e., no Dutch reference values are available at this time) and Dutch reference values for V1.0 Depression and V1.0 Anxiety [48] and V1.0 Sleep Disturbances [49]. The Cognitive Failures Questionnaire scores were compared with Dutch reference values [43]. Median, range, and frequencies were used to evaluate ratings of the cognitive tasks (Phases I and II) and the cognitively enriched walking sessions in general (Phase II). For Phase II, the best (i.e., highest median and smallest range) and least (i.e., lowest median and widest range) positively evaluated cognitive tasks were discussed. Furthermore, the mode was calculated for all cognitive tasks and rating scales of Phase I and Phase II. These values are not reported in the paper as they do not differ much from the median (which we do report), but can be found as Supplementary Files 3 (rating of cognitive tasks—Phase I), 4 (rating of cognitive tasks—Phase II), and 5 (rating of cognitively enriched walking sessions).

#### 2.7.2. Qualitative

Data from the open-ended questions from the walk-along interviews (Phase I) were coded by two researchers independently. Any disagreements were discussed with a third researcher to reach consensus coding. This content analysis was performed in SPSS. In the results section, counts are provided to quantify the number of times a (sub)code was used. The final coding tree can be found in Table 3 and was drafted deductively based on the questions asked during the walk-along interview and adapted inductively (i.e., while coding).

Qualitative data from the program evaluation questionnaires (Phase II) were briefly summarized. The audio recordings of the focus groups were transcribed verbatim and coded by two researchers using QSR Nvivo qualitative data management software. In case of disagreement, a third researcher was involved to reach consensus. This thematic analysis approach aimed to gain a deeper understanding of the feasibility and acceptability of and suggestions for improvement of the cognitively enriched walking intervention. The coding tree (Table 4) was drafted deductively based on the interview guide used for the focus groups and adapted inductively. The same coding tree was used for the data from both focus groups.

## 3. Results

### 3.1. Participant Characteristics

#### 3.1.1. Phase I

A total of 176 people expressed willingness to participate in Phase 1, of which 13 participants (7%) did not meet the inclusion criteria. Thus, 163 older adults participated in a total of 85 walk-along interviews (i.e., there were individual walk-along interviews and walk-along interviews in pairs). The participants were on average 75.13 years old (SD = 6.26, range: 65–89), 58% was female, and 42% was male. Furthermore, 36% (*n* = 59) of the sample obtained a higher education degree, 40% (*n* = 65) obtained a secondary education degree, 18% (*n* = 30) obtained a primary education degree, and 3% (*n* = 4) obtained no degree. For the other 3% of participants (*n* = 5), the latter information was missing.

#### 3.1.2. Phase II

Twenty-two participants were enrolled in the group-based phase of the study and met the inclusion criteria, of which three dropped out (13.6%) before starting the intervention because of health issues (*n* = 2) or an acute family event (*n* = 1). Nineteen participants (i.e., nine in Leuven and ten in Ghent) started the three-week pilot intervention. Participants were on average 73.63 years old (SD = 5.51, range: 65–88 years), 63% of the sample was female, and 37% was male. Furthermore, 84.2% of the sample (*n* = 16) obtained a higher education degree, and the remaining 15.8% (*n* = 3) obtained at least a higher secondary education degree. Other sample characteristics can be found in Table 5.

Prior to the start of the intervention, the proportion with high, moderate, and low PA levels was 47.4% (*n* = 9), 36.8% (*n* = 7), and 15.8% (*n* = 3), respectively. The mean total CFQ score of the participants (M = 34.6; SD = 12.5) was within the normal range (21–44), which is based on the sample of the Maastricht Aging Study (*n* = 1358) [43]. The mean T-scores on PROMIS short forms Depression (M = 51.0; 7.5), Anxiety (M = 51.8; SD = 8.0), Sleep Disturbances (M = 48.3; 10.0), and Social Isolation (M = 42.6; 9.1) are within one standard deviation of the Dutch reference values for Depression (M = 49.9; SD = 10.1) [48], Anxiety (M = 49.6; SD = 10.0) [48], and Sleep Disturbances (M = 49.7; SD = 9.8) [49] and the US reference values for Social Isolation (M = 50; SD = 10) [50].

### 3.2. Quantitative Results

#### 3.2.1. Phase I

Thirty-one of the 32 cognitive tasks that were evaluated had median rating scores of 3, 3.5, or 4 on the rating scale for *feasibility* ranging from “Totally disagree (0)” to “Totally agree (4).” Only the cognitive task “Problem solving” received a median rating score of 2 for feasibility. In terms of *enjoyability*, all cognitive tasks obtained a median score of 3, 3.5, or 4, on a scale ranging from “Totally disagree (0)” to “Totally agree (4).” There was variability in perceived feasibility (Table 6) and enjoyment (Table 7) as indicated by the individual scores ranging from 0 to 4.

#### 3.2.2. Phase II

*(1) Evaluation of the Cognitive Walking Sessions*. No session received a score below 5 on a rating scale of 0 “Not good at all” to 10 “Very good,” on the question “How would you evaluate this cognitively enriched walking session?” Furthermore, ≥65% of the participants for all walking sessions rated the walking sessions with a score of 7 or higher. Median scores for the cognitively enriched walking sessions ranged from 7 to 10 on a total of 10 (Table 8).

*(2) Evaluation of Cognitive Tasks*. Median scores and ranges for all cognitive tasks on the different rating scales (ranging from “Totally Disagree (0)” to “Totally Agree (10)”) are presented in Table 9, sorted according to median scores on the difficulty rating scale. Median scores of lower than 5 were only obtained for the *difficulty* of the cognitive tasks (i.e., range of medians: 3–9). Median scores of 5 or higher were obtained for *feasibility* (i.e., range of medians: 8–10), *enjoyability* (i.e., range of medians: 7–10), *meaningfulness* (i.e., range of medians: 7–10), *challenge* (i.e., range of medians: 6–10), *interaction* (i.e., range of medians: 5–10), *competition* (i.e., range of medians: 5–9.5), *appropriateness for combining them with walking* (i.e., range of medians: 7–10), *appropriateness for the age group* (i.e., range of medians: 6–10), *perceived positive influence on the brain* (i.e., range of medians: 5–10), and *clarity of the instructions* for the cognitive tasks (i.e., range of medians: 8–10). As shown by the ranges of the individual scores, there was a relatively large variability in scores (Table 9). The largest variability was found for the *difficulty* and *competition* ratings, with individual scores ranging from 0 to 10.

“Music” was the least *difficult* task (median (M) = 3, range = 1–9), while “Quest with clues + remember the route” was the most *difficult* task (M = 9, range = 7–10). The least *feasible* task was “I spy” (M = 8, range = 5–10) and the most *feasible* task was “Obstacle walk” (M = 10, range = 7–10). The least *enjoyable* task was “I spy” (M = 7, range = 5–10), and the most *enjoyable* task was “Music” (M = 10, range = 6–10). The least *meaningful* task was “I spy” (M = 7, range = 5–10), and the most *meaningful* task was “Immediate recall” (M = 10, range = 7–10). The least *challenging* task was “I spy” (M = 6, range = 3–10), and the most *challenging* task was “Quest with clues + remember the route” (M = 10, range = 7–10). The least *interactive* task was “Immediate recall” (M = 5, range = 0–10), while the most *interactive* task was “Quest with clues + remember the route” (M = 10, range = 6–10). The task with the least *competition* was “Immediate recall” (M = 5, range = 0–10), while the task with the most *competition* was “Quest with clues + remember the route” (M = 9.5, range = 3–10). The tasks least *appropriate for combining with walking* were “Hidden word” and “Buzz it + problem solving” (both M = 7, range = 5–10), while the task most *appropriate for the combination with walking* was “Quest with clues + remember the route” (M = 10, range = 7–10). The task found least *appropriate for the age group* was “I spy” (M = 6, range = 3–10), while the task found most *appropriate for the age group* was “Quest with clues + remember the route” (M = 10, range = 7–10). The task in which the *perceived positive influence on the brain* was the least was “I spy” (M = 5, range = 3–10), and the task in which the *perceived positive influence on the brain* was the greatest was “Quest with clues + remember the route” (M = 10, range = 7–10). The least clear *instructions* were given for tasks “Ball games” (M = 8, range = 0–9), while the most clear *instructions* were given for the task “Immediate recall” (M = 10, range = 7–10).

*(3) Attendance*. Of the 19 participants that started the three-week pilot intervention, 12 (63%; *n* = 8 in Leuven and *n* = 4 in Ghent) completed all six cognitively enriched walking sessions. The average attendance was 87.7% (SD = 20.6%), which corresponds to 5 out of 6 sessions (SD = 1 session). Reasons for not attending a session were illness, conflicting appointments, and self-isolation because of COVID-19.

### 3.3. Qualitative Results

#### 3.3.1. Phase I

The content analyses of responses to the open-ended questions during the walk-along session indicated that that good weather conditions (*n* = 13), letting the walk take place in a quiet environment without many obstacles (*n* = 11), and the opportunity for social or group interaction (*n* = 3) were important for a positive evaluation of the walk. Reasons for a negative evaluation of the walk were bad weather conditions (*n* = 30) and having difficulty walking long distances because of physical discomfort (e.g., knee pain or needing help to walk) (*n* = 5).

When assessing specific cognitive tasks itself in terms of enjoyment and feasibility, only having the feeling that the tasks stimulates self-efficacy by enactive mastery (*n* = 2) and finding the difficulty level of the task just right (*n* = 2) were mentioned to be a reason for a positive evaluation of a given cognitive task.

Perceiving the cognitive task as too difficult (*n* = 25) or too easy (*n* = 22) and not finding the cognitive task suitable to perform while walking (e.g., because one cannot relax or talk enough) (*n* = 13) were the most common reasons for a negative evaluation of a cognitive task. In addition, not seeing the benefit of the tasks or feeling like the tasks were unnecessary or not useful (*n* = 8), the absence of competition (*n* = 8), the feelings of shame (e.g., because other people watch you doing the task) (*n* = 6), finding the task too boring (*n* = 5), and finding the task too childish or not appropriate for older adults (*n* = 5) were also mentioned frequently as reasons for a negative evaluation of a cognitive task. Lastly, another reason for not liking a task or not finding a task feasible was not feeling safe because of fall risk while performing the task (*n* = 9), because of traffic (*n* = 3), or because of uneven ground (*n* = 1).

#### 3.3.2. Phase II

*(1) Open-Ended Questions*. Mostly information about practical aspects of the walks came up (i.e., starting place or weather conditions). Getting to know each other and social interaction were indicated as aspects participants liked the best about the sessions. The walking speed (i.e., too slow), walking distance (i.e., not far enough), weather conditions (i.e., rain or cold), and unclear instructions about the cognitive tasks were the main aspects participants mentioned to like least about the sessions.

*(2) Focus Groups*. All participants of the walking group in Leuven (*n* = 9) were present during the focus group, while only five out of ten participants of the walking group in Ghent were present during the focus group. Results for the different themes that were covered during the focus groups are described below.

- The Content of the Cognitive Tasks
- Overall, most participants perceived the cognitive tasks to be less difficult than they had expected. About half of the participants agreed that the cognitive tasks were too easy for them and even felt as if their capacities were underestimated. Other participants perceived part of the tasks as difficult and part of the tasks as not difficult at all. Only one participant mentioned that some of the tasks were too difficult, but that he tried to participate, nevertheless. One participant suggested making some tasks more difficult by extending the recall phase in memory exercises (e.g., repeating the recall at the beginning of the next walking session). She would also like more attention to be paid to the strategies they used to solve cognitive tasks, in order to get the opportunity to learn from each other. Other participants would like to have the option to choose a certain level of difficulty (e.g., different walking groups based on difficulty or different levels of difficulty for each cognitive task).

*“But for me it was too difficult. I have often told myself ‘I can't keep up with the others, that's that.'” [researcher “So sometimes it was too difficult for you. Would you prefer easier tasks?”] “No, no, I tried to participate. That's no problem for me.” (G006, male, 88 y)*

  Most of the participants reported that it was feasible to perform the cognitive tasks while walking. However, they mentioned that sometimes they automatically stopped walking to chat or perform a task and that motivational encouragement of the coach was needed to keep them walking. It was also mentioned that tasks that included writing and were inherently walking-unrelated (e.g., “Mental Arithmetic”) were not or less feasible to perform while walking. Tasks needing communication or interaction between participants were, according to these participants, the most feasible and fun tasks. In order to make the tasks more feasible to perform while walking, some participants suggested including more verbal tasks instead of tasks that need reading or writing. Furthermore, they preferred tasks to reflect daily life, or to make use of more relevant stimuli (e.g., remembering the age of the other group members).

*“I think that all tasks in which you communicate with each other are feasible, you are walking and that way you get to know the person next to you a bit better and you are observing or doing other things at the same time.” […] “I find this [i.e., mental arithmetic] very abstract. You are walking in nature and combining it with something like that, that was not natural to me.” (L001, female, 70 y)*

*“A bit closer to reality and what is interesting to me […]. That way, it is less artificial than other things to remember.” (L006, male, 73 y)*

  There was a distinction between perceptions of inter- and intra-individual competition. Regarding inter-individual competition (e.g., trying to recall the most words, or to be the quickest to solve a riddle), opinions were divided in both groups. Some participants mentioned to be very reluctant to inter-individual competition, while others mentioned that they liked to compete with others. On the other hand, it was mentioned that some like intra-individual competition, namely, monitoring the evolution in their own performance or comparing themselves to age-related norms. Participants who liked either inter- or intra-individual competition both mentioned that this could be a motivator for them.

*“For me, I don't need competition [i.e., with others], but I need competition with myself.” [Referring to another participant saying, “I have always been competitive, also in sports.”] “Yes, me too, but not to compare with someone else. For myself.” (G004, female, 65 y)*

*“I would include inter-individual competition, because the ones that don't mind competition, will not mind losing.” (L004, female, 71 y)*

(b) The Participants' Experience of the Cognitive Walking Sessions
  Most of the participants were not satisfied with the difficulty of the physical aspect of the cognitive walks. They preferred a faster walking speed and a longer walking distance. During the group walks, approximately three kilometers were walked in 60 minutes.

*“For me, the walking speed was very slow. The distance we covered was limited. I think we are all people that walk a lot, and this was not challenging enough. […] If you respond to a call for participants for this kind of study, I think you are automatically interested in walking. And someone our age, who isn't physically active, will probably not participate in this study.” (L001, female, 70 y)*

  Feasibility of the program was evaluated positively by the participants. However, as mentioned above, they sometimes forgot to keep walking (e.g., because they were talking or performing cognitive tasks) and would thus need more encouragement to continuously keep walking. One participant mentioned that he lost awareness of and connection with nature because he was focused on the cognitive tasks.
  Furthermore, a participant mentioned social interaction as a motivator to do sports. Others also mentioned that they liked the fact that this was a group program. Some indicated that the group setting works encouraging. However, this can also imply that participants will put less effort in solving the cognitive tasks themselves.

*“I would certainly choose to keep it in group, because if you're alone, you are more prone to say, ‘I can't do that, I don't know that,' and now you could say ‘he [the other] will know it, it's okay.' So, you were more relaxed while participating. Because you keep in mind ‘if I don't know it, he will.'” (L005, male, 66 y)*

(c) Guidance of the Cognitive Walks
  It was mentioned that the instructions for the cognitive tasks provided by the coach were not always clear, which made it sometimes difficult to perform the cognitive tasks correctly.
  For most of the participants, the framing of the cognitive tasks was very important. They wanted to know why they were doing certain tasks. Although an explanation about why each cognitive task was important was already given, most participants of both groups would like more background information, also about the general aim of the program.

*“I want to know why we do every task.” […] “Like, is it to keep us busy, or is it evidence-based?” (G004, female, 65 y)*

  Participants indicated that it is important that the coaching style should be sufficiently encouraging to make them perform the cognitive tasks the right way and to keep walking. A humoristic, friendly, and inclusive (i.e., trying to engage all participants) coaching style was liked by the participants. However, they did not like it when the coach would try to solve the cognitive tasks for them. The participants mentioned that they do not mind the age of the coach. Other preferred characteristics of the coach were not mentioned.
(d) Characteristics of the Cognitively Enriched Walking Program
  Most of the participants agreed with the proposed duration of one walking session, which is 60–90 minutes. Some participants, however, mentioned that they would like longer walking sessions, depending on the season (e.g., in winter 60–90 minutes is enough, in summer longer walks are preferred). The participants did mention that a duration of less than 60 minutes would be too short and not worth the effort of coming to the starting point and organizing a group walk.
  Opinions were divided about the frequency of the cognitive walking sessions when the program would be held for a longer time period (i.e., six or nine months). A substantial part of the participants mentioned that two times a week was too much, and that one session a week was preferred. Others agreed with two sessions a week, or preferred only one, but a longer session. One participant mentioned that she would even like three sessions a week. A reason for preferring one session a week (i.e., coming from one participant) was that she already has too many other activities, and another participant mentioned the long distance from his living place to the starting point of the sessions. Living near the starting point, the group dynamics (i.e., a “nice” group), and spontaneous social activities after the walking sessions (e.g., having a coffee together) were mentioned by one participant as reasons for wanting to engage in two sessions a week. Because of the participants' reluctance to attend two or more sessions a week, which was advised by experts in the previous stage of this project [33], we asked the participants' opinions on the possibility of individual (nonsupervised) cognitive walking sessions to supplement the group sessions. Most participants were enthusiastic about this, as they could fit this into their already existing habits. They did mention that they would like the cognitive tasks to be low threshold (e.g., simply repeating a task that was performed during a cognitive walking session in group) and real-life reflecting and to be able to choose to do these sessions alone or in company. However, some participants were doubtful about their long-term adherence to these individual sessions.
  Regarding the duration of the program, some participants were reluctant to engage for a longer period (i.e., six or nine months). Some participants mentioned that this is too long, and others mentioned their other activities as a reason for not being able to engage themselves for a longer period.

*“Driving here by car was okay for two times a week for three weeks. But for a longer period, I wouldn't do that.” (L005, male, 66 y)*

(e) Organization of the Program
  While some participants preferred communication through e-mail, others preferred contact via telephone. Some participants mentioned that they liked the *WhatsApp* group (i.e., communication app on smartphone) that was organized for the group in Leuven to facilitate communication between coach and participants. Regarding safety, the participants in both groups mentioned that a place with few cyclists, joggers, or other traffic feels safe for them. Almost all participants preferred to walk in nature instead of in the city center. The availability of parking spaces was mentioned as an important factor when choosing the location for the cognitive walking sessions; public transport or other modes of transportation were not mentioned. Weather conditions were mentioned as an important factor by some participants. While the participants preferred not to walk in the rain, some of them indicated that they did not mind it, noting that it is possible to dress accordingly.
(f) Composition of the Walking Groups
  Because of different physical fitness levels, the ideal walking speed was not the same for everyone. Some participants did not experience this as a problem, other participants mentioned that it is important for all members of a walking group to have a similar walking speed (i.e., so no one has the feeling they have to walk too slow or too fast). It was also suggested that it might be better to have different groups for different fitness levels. The participants mentioned that the age of the group members does not matter as some older individuals are more fit than younger ones. Regarding the group size, all participants preferred a group of eight to twelve people and they thus agreed that the size of the walking groups they were in now was optimal.

## 4. Discussion

The aim of this study was to evaluate the feasibility and acceptability of the cognitively enriched walking program for community-dwelling older adults.

In general, the cognitive tasks showed adequate feasibility and enjoyability in both the walk-along interviews (Phase I) and the group-based program (Phase II). Median scoresof 2-4 for feasibility and 3-4 for enjoyability on a total of 4 were obtained in Phase I. In Phase II, median scores of 8-10 for feasibility and 7-10 for enjoyability on a total of 10 were obtained. Also the meaningfulness, challenge, interaction, competition, appropriateness for combining with walking, appropriateness for the age group, perceived positive influence of the brain, and clarity of the instructions of the cognitive tasks were overall positively evaluated in Phase II with medians ranging from 5 to 10 on a total of 10. Furthermore, the cognitively enriched walking sessions were positively evaluated with medians of 7–10 on a total of 10. Moreover, in Phase II, an average adherence of 87.7% (or 5 out of 6 sessions) was obtained, which confirms the feasibility and acceptability ratings. The cognitive tasks, as well as the cognitively enriched walking program, are thus evaluated to be feasible and acceptable. This suggests that further evaluating the effects of the group-based cognitively enriched walking program is likely to be achievable in the real world. However, results also showed that some adaptations need to be made to the cognitive tasks as well as to the group-based program itself to be ready for execution and evaluation of its effects by means of a randomized controlled trial (RCT).

### 4.1. Cognitive Tasks (Phases I and II)

Most participants provided positive ratings for the cognitive tasks in terms of enjoyment and acceptability. However, cognitive tasks during which writing was needed (e.g., “Mental arithmetic”) were changed from written to verbal tasks in the subsequent RCT study. This was done to meet the suggestion of participants that tasks needing social interaction or verbal discussion instead of tasks that need writing or reading are better suited to perform while walking in group because they interfere less with the natural flow of the walk. Furthermore, as a response to the participants of Phase II mentioning they like tasks better when they make use of stimuli relevant to their daily life, we emphasized this in the RCT manual for coaches by adapting the examples we give. For instance, for the cognitive task “List learning,” we suggested trying to remember things on the shopping list of the participants, in addition to trying to remember street names they encounter during the walk.

Notably, low median scores (i.e., lower than 5 on a total of 10) were obtained only for the difficulty of the tasks, showing that most of the tasks were not difficult (or too easy) according to most of the participants. This was also reflected in the results of the focus groups after Phase II of this study, in which some participants even mentioned that they felt as if their capacities were underestimated. Moreover, participants of Phase I mentioned tasks being too easy as a reason for a negative evaluation of a given task. It is however important to note that there was a large variability in individual evaluations of difficulty, and the sample of Phase II was highly educated and generally reported few subjective cognitive failures. Furthermore, Gheysen et al. [24] suggested that adequate cognitive challenge is important to obtain cognitive effects. It is consequently of utmost importance to differentiate during the cognitive walking session (i.e., to make it possible to adapt the cognitive task to meet each participant's individual capacities). Although efforts to make this possible were already made by describing different variations of the cognitive tasks in the manual for the coaches, even more specific options to tailor cognitive tasks were provided in the manual for the coaches to be used in the RCT. This could imply that it is necessary to perform cognitive tasks in smaller groups, based on the individual capacities of the participants. However, to ensure the core concepts of the cognitive tasks remain the same even when tailoring the difficulty level (e.g., by having to remember more or less words or facts), the manual for the RCT also indicated which are the “basic building blocks” for each task. The coaches were instructed not to change these aspects. For example, the basic building blocks of the cognitive task “Facts and titbits” are the following: (1) exchanging information with other participants while walking, (2) memorizing new information while walking, and (3) retrieving the newly acquired information from memory while walking.

Furthermore, although there was no opposition against competition among the participants (i.e., during the focus groups, Phase II), there was a large variability in perceived competition ratings for all cognitive tasks (i.e., range of individual scores of 0–10). Thus, older adults might just have different perceptions and preferences with regard to competition. Previous research showed that competition can be a motivator for PA participation [51], but that women may be less likely to prefer competition than men [52]. Hence, competition will continue to be included on an occasional basis, as recommended by Marent et al. [33]. Specifically, the coaches in the RCT were advised to include competition carefully, to prevent conflicts or a bad atmosphere and to make intra-individual competition possible.

One cognitive task, “N-back,” that was evaluated during the walk-along interviews, was left out when evaluating the group program. The researchers decided upon this based on the findings of Marent et al. [33], the experience of the researchers that is was not easy to make this task enjoyable to perform in group, and the relatively low median enjoyability score obtained in Phase I of this study. Furthermore, it is important for the practical application of the program to include tasks that are perceived to be enjoyable. This is in line with literature showing that enjoyment is a reason for participation in PA for older adults [53, 54]. This task was thus also left out when evaluating the effectiveness of the program.

### 4.2. Cognitively Enriched Walking Program (Phase II)

Overall, the cognitively enriched walking sessions were positively evaluated (i.e., medians of 7–10 on a total of 10 and no score below 5). This is encouraging, as recent research has considered acceptability as an important factor that may influence adherence and contribute to the effectiveness of the intervention [55–57]. Furthermore, enjoyment could motivate older adults to undertake and maintain PA [58].

However, participants did not perceive the walks as not physically challenging enough and most of the participants preferred a higher walking speed and longer walking distance. A possible explanation might be that participants were not given clear information about the expected walking speed and distance prior to signing up. To set more realistic expectations for the upcoming RCT study, it was communicated during recruitment that walking distance during a cognitively enriched walk of 60 minutes would be three to five kilometers. Important to note, however, is that most of the participants in Phase II of this study were highly physically active at baseline. This could be explained by the well-known self-selection bias in PA trials, in which people who enroll are typically already physically active. For example, in an RCT by Sipilä et al. [22] focused on people not meeting the physical activity guidelines, 2,767 people were assessed for eligibility of which 806 (29%) had to be excluded because they were too physically active. Likewise, van Uffelen et al. [59] did not manage to include people with low PA levels in a one-year community-based PA program to improve cognitive function and therefore also included more active people and adjusted the program to cater for people of different PA levels. The baseline PA levels of Phase II participants may have influenced their opinion about the preferred walking speed and distance. Given that it is a group program, differentiating in walking speed or distance on an individual level is very challenging. When implementing this program on a larger scale (i.e., after the effect evaluation), different walking groups for different levels of fitness levels could be created. Of course, it is important to try to physically challenge everyone sufficiently as earlier work already showed the importance of progressive increase of difficulty of physical exercise to induce cognitive effects [60]. Furthermore, it became clear that participants tended to slow down or even stopped walking while performing the cognitive tasks. This is consistent with the finding that gait speed is negatively impacted by dual-tasking in healthy older adults, as shown by a meta-analysis including 22 studies [61]. Given the observations made in this study as described above, the following elements were added to the manual for the RCT: (1) as the aim of the intervention is to simultaneously perform cognitive tasks while walking, the importance of keeping the participants moving was emphasized in the manual and this was also highlighted during the program education session for the coaches in the RCT; (2) ensuring everyone could walk at a sustainable walking speed while still being able to join the group-based walking program, an extra emphasis was placed upon the individual differentiation in walking speed; participants walking faster could, for example, be instructed to proceed to a certain point on the route at their own speed and to return to the group when reaching this point; (3) ensuring the intensity of the activity increased, the coaches were instructed to progressively increase the walking speed over the intervention duration of six months. Furthermore, we recruited certified walking coaches for the RCT (like in the pilot study), who have undergone a formal training. They therefore have the necessary competences to ensure all participants can maintain a walking speed which is sustainable for them, and demonstrate proficient skills in this regard. Altogether, the manual for the coaches to be used in the RCT was extended based on the observations made in this pilot study. As described above, basic building blocks and options for variation in difficulty for all cognitive tasks and considerations for the physical part of the intervention were included in the manual. In addition to those changes, the manual was extended to incorporate information regarding the aim of the intervention, the basic principles of the cognitively enriched walking intervention, the practical organization of the sessions and strategies for ensuring a safe walking environment.

Regarding the practical aspects of the program, it was agreed upon by participants that a duration of 60–90 minutes for one cognitive walking session was ideal, especially keeping in mind the frequency of two group sessions a week. Some participants preferred longer sessions, but with a frequency of only one group session a week. Experts advised having at least two sessions a week to induce effects on cognitive functioning [33]. A compromise solution could involve having two group sessions a week supplemented with one home-based individual session. This is in line with another combination of PA and cognitive training by Sipilä et al. [62], who also supplemented two group exercise sessions with a home exercise program and home-based cognitive training. This individual session was included in the subsequent RCT study by providing participants with “practice cards” that could be used when going for a walk on their own or for instance with their partner, (grand)children, or friends. These cards included the cognitive tasks performed during the group walks, adapted to make it possible to perform them alone, without needing extra equipment or a coach to supervise. With respect to the total duration of the program, participants were reluctant to engage themselves for a longer period (i.e., 6 or 9 months), mainly because combining it with other activities would not be feasible in the long run.

The supervision of the sessions by a coach was considered a positive aspect of the program by the participants. Regarding the influence of supervised (i.e., a researcher or instructor is present to give instructions) versus unsupervised interventions (i.e., the participant performs the intervention without a researcher or instructor being present), Gavelin et al. [23] did not find a moderating effect of supervision for PA + CA interventions. However, only 4 studies did unsupervised training (versus *n* = 37 for supervised training), and thus these results should be treated with caution [23]. Framing the cognitive tasks is important as the participants mentioned they want to understand why they are doing certain tasks. This can support the basic psychological need of autonomy, as described in the self-determination theory (SDT), which can positively impact exercise participation [63]. The group setting was also mentioned as an advantage of this program, as social interaction could be a motivator to be physically active. This is in line with recent research indicating that social factors such as spending time with others [52] or having an exercise partner [58] might improve participation in PA interventions and therefore lead to better results of these interventions (i.e., greater improvements in PA levels). Accordingly, Zhu et al. [25] found larger effect sizes for the improvement of cognitive functioning in group-based PA + CA interventions, compared with individual or mixed interventions. Given the importance of the social aspect, a measure for social support and loneliness was included when evaluating the effects of the cognitively enriched walking program.

### 4.3. Most Important Factors of a Feasible and Acceptable Cognitively Enriched Walking Program

The most important factors for a feasible and acceptable real-life cognitively enriched walking program, identified in this study, are as follows: (1) the program should be sufficiently challenging for every participant both cognitively as well as physically, (2) social interaction should be encouraged as this could be a motivator, (3) solving of the cognitive tasks should mainly be verbal instead of written, (4) cognitive tasks should make use of stimuli reflecting daily life, (5) the rationale of performing the cognitive tasks should be explained, (6) cognitive tasks should be conducted in group, (7) the cognitively enriched walking program should not have more than two group sessions a week, and (8) the cognitively enriched walking program should be supervised by a trained coach.

### 4.4. Strengths and Limitations

This study had several strengths. Conducting a pilot study before the start of anRCT in a large sample is a strength, since pilot testing often provides ideas and approaches that may not have been foreseen before conducting the effect evaluation study (RCT), and may result in adaptations or redesign of the intervention that may increase the chances of finding effects in the RCT. This is especially relevant when translating interventions from controlled to real-life settings, as feasibility and enjoyability will influence participation rate and adherence. Secondly, the mixed-methods design (i.e., using both quantitative and qualitative data) is a strong design which allowed a deeper investigation of the underlying reasons for the feasibility and acceptability ratings and the experiences of the participants, making it possible for the researchers to better adjust the program to the specific needs of the target group. Also, doing individual walk-along interviews had several benefits as it allowed a one-on-one conversation with the participant, which limited potential social or group biases. Furthermore, these interviews were performed *during* the walk and right after the moment of doing the cognitive tasks which minimized potential memory or recall biases. Finally, although breaking the study apart into two phases was an unforeseen amendment to meet COVID-19 regulations, it enabled us to gain detailed insight in potential differences between the experience of performing the cognitive tasks individually at one time point versus performing the program (i.e., the combination of 2-3 cognitive tasks performed while walking) in group for a longer period, but also to see similarities in the feedback that was given which added more certainty to the answers being representative for the general opinion of the target group. Moreover, the two-phased design made it possible to make further adjustments to the program before conducting it in a group.

There were also some limitations to this work. First, Phase II of the study (i.e., group walks) was conducted in a limited sample (*n* = 19) which might hinder generalizability of the results to the wider population of healthy older adults. However, many different guidelines for sample sizes for pilot studies exist, indicating that sample sizes of 10 [64] to or 30 or more [65] are needed. Although Teresi et al. recommend a sample size of 30 or less or 30 or more when using qualitative or quantitative methods, respectively [65], we are not aware of specific guidelines for sample size in a pilot study using mixed-method to evaluate the feasibility and acceptability of an intervention. Furthermore, Teresi et al. emphasized that the sample size should be based on practical considerations, such as budgetary and time constraints [65]. For the present study's aim, which was to evaluate feasibility and acceptability—not to estimate effect sizes or make between-groups comparisons—and taking into account the cognitive tasks were already evaluated by 163 individuals in Phase I, the sample size of *n* = 19 for Phase 2 allowed us to gather meaningful data using both quantitative and qualitative methods. This comprehensive evaluation allowed us to detect potential issues in feasibility and acceptability of the program before finalizing the program manual and start the recruitment phase for the RCT. This sample was also highly educated and highly physically active, which implies that the results and suggestions that were made are less representative for people with a lower educational degree or a lower PA level. Second, we excluded participants based on self-report of having a cognitive disorder and did not use a screening test to rule out cognitive impairment in either phase of the study. Third, in both phases, interviewers and coaches only received a short training which might have impeded the standardized execution of the program and instructions to the cognitive tasks. The limited training of the coaches may however add to the ecological validity of our findings. Furthermore, in Phase I, the walk-along interviews were conducted by several interviewers who only received a document with instructions and no individual training by the research team, which might have caused variability in the way cognitive tasks were explained and, consequently, experienced by the participant. Fourth, the open-ended questions completed by participants in Phase II were written in positive tones, which might have lead participants to answer more positively.

Finally, as with most research conducted during 2020-2021, last-minute changes had to be made in the study protocol because of the restrictions that were imposed by the Belgian government to prevent the spread of COVID-19. As mentioned in the methods section, this resulted in a two-phased design, but it might also have impacted the willingness to participate in a study with total strangers. As such, individuals that were more cautious, were at a higher risk of coronavirus complications, or lived with a family member that was at higher risk, might not have participated. This was also the reason why bachelor students recruited and interviewed participants of their own network for the walk-along interviews (Phase I), which may have caused a bias towards more positive evaluations because participants might not have wanted to disappoint the interviewer. Furthermore, due to postponing the group-based testing because of COVID-19 regulations and because of our funding timescale, there was no other option than to conduct Phase II in the colder months of the year. We however see this as a strength since this adds to the ecological validity and the results indicate that, also in the colder months, this program is feasible and acceptable. Nonetheless, it needs to be noted that Belgium has a mild climate all year round and that an outdoor walking program may be less feasible in regions with much colder climates.

### 4.5. Future Research Directions

Given the need for accessible, low-cost programs for the prevention of cognitive decline and promotion of optimal cognitive aging, real-life interventions like this one are increasingly relevant. Although research is growing in the area of healthy aging, real-life programs will only be adopted when feasible and acceptable to the end-users. As shown by our results, also contextual factors such as weather or walking environment are important in real-life programs, which is not the case in controlled settings. More real-life programs should thus be developed in co-creation with their end-users and include pilot testing in order to be able to compare or confirm our findings. Furthermore, as this study sample of healthy older adults was highly educated and physically active, other, more vulnerable groups of older adults should be included in future studies as other factors might be more important for them. Additionally, these findings are only of relevance to healthy older adults, and thus an intervention for older adults with cognitive decline (i.e., mild cognitive impairment or dementia) should be adapted to the needs of this specific study population. Lastly, as this study focused on feasibility and acceptability, it did not provide data on whether or not the cognitively enriched walking program can mitigate cognitive decline. Therefore, further interventional research to evaluate the effectiveness of this real-life group-based cognitively enriched walking program is warranted.

## 5. Conclusion

Overall, this study provides evidence for the feasibility and acceptability of this group-based cognitively enriched walking program for community-dwelling older adults. The most important factors for a feasible and acceptable real-life cognitively enriched walking program are as follows: (1) the program should be sufficiently challenging for every participant both cognitively as well as physically, (2) social interaction should be encouraged as this could be a motivator, (3) solving of the cognitive tasks should mainly be verbal instead of written, (4) cognitive tasks should make use of stimuli reflecting daily life, (5) the rationale of performing the cognitive tasks should be explained, (6) cognitive tasks should be conducted in group, (7) the cognitively enriched walking program should not have more than two group sessions a week, and (8) the cognitively enriched walking program should be supervised by a trained coach.

These results warrant future research to establish the effectiveness of this program. In addition to the actual results of this study, the employed methodology is relevant for researchers and practitioners planning to pilot test the acceptability and feasibility interventions that have been adapted for use in real-life settings.

## Figures and Tables

**Table 1:** Overview of cognitive tasks.

Cognitive task	Brief description
Facts and titbits	Participants share facts and titbits with each other. At the end of the walk, “Guess who” is played: a fact or titbit is shared and participants must try to remember whose it was
Quest with environmental clues	Participants try to find the correct route by means of environmental clues
Awareness	A sort of mindfulness exercise, to be more aware of their environment (e.g., five senses exercise)
Spotted	Participants look out for certain aspects in the environment (e.g., flowers, trees, and buildings)
Opinions	A conversation is held about socially relevant topics. Participants are encouraged to form an opinion and share this opinion in a constructive manner.
Notice and remember symbols	Participants must look out for symbols placed on the route and try to remember these symbols and the location they were placed at
Quiz	Answering questions (e.g., about the environment they encountered during the walk and about historical facts)
Plan the route	Participants plan their own route
Quest with riddles	By solving riddles, participants can find out the correct route
Hidden word	The participants take turns in describing a certain word. The others must guess the word as fast as possible.
Word fluency^a^	A particular letter or category is chosen after which the participant tries to name as many words as possible, starting with the chosen letter or belonging to the chosen category
Problem solving	Participants try to solve riddles
Word associations	In this task, participants try to connect words that are associated with each other
Remember the route	Participants try to remember the route they followed
I spy	One participant chooses an object in the environment, of which others have to guess what it is by asking questions
A new language	Participants teach each other words or sentences in a (to them) new language or dialect
Buzz it	A funny question is asked, after which participants try to give their funniest answer
Story telling	Together, participants create a story by adding a sentence one by one
Geocaching	This task consists of a quest for a treasure. The location of the treasure is given at the start of the session, after which participants must try to find the fastest route to the location of the treasure.
Serial subtraction task	Participants count down from, e.g., 100, by, e.g., sevens
Memory techniques	Participants learn a memory technique that can help to remember certain words (e.g., Loci-method)
Music	Old songs are played, and participants try to remember the lyrics
Obstacle walk	An obstacle walk is held
Order of daily activities	Every participant receives an action that is part of a daily activity. Altogether, participants must put their actions together and find the correct activity.
Mental arithmetic	Calculations are made (e.g., simply counting and making sums)
The alphabet	Participants recite the alphabet in the correct or reverse order or skip two letters at a time
Choreography	Participants perform extra movements while walking (e.g., two steps forward and five steps backward)
Immediate recall	Lists are repeated during the walk (e.g., street names and house numbers). Participants try to repeat the list in the same, reverse, or alphabetical order.
N-back	A list of letters, words, or numbers is read; participants must respond with a predefined signal (e.g., shouting “yes”) when a letter, word, or number was already previously mentioned
List learning	A variety of possible lists are studied, and participants try to repeat them at the end of the walk
Stimulus-response	Participants must respond to a predefined stimulus in a certain way
Ballgames	Participants must remember the order in which the balls are thrown. The complexity can be increased by using more than one ball or adding rules.

*Note*. ^a^“Word fluency” is the same cognitive task as “Words starting with a particular letter” in the article of Marent et al. [33].

**Table 2:** Themes of cognitive walking sessions and cognitive tasks (Phase II).

Theme of walking session	Cognitive tasks
(1) Session A: getting to know each other *(Leuven and Ghent)*	(1.1) Facts and titbits
	(1.2) Spotted

(2) Session B: family and friends *(Leuven)*	(2.1) Immediate recall
	(2.2) Notice and remember symbols
	(2.3) Memory techniques

(3) Session C: 70s 80s 90s *(Leuven)*	(3.1) Mental arithmetic
	(3.2) Music
	(3.3) Word associations

(4) Session D: hedonists *(Leuven)*	(4.1) Plan the route
	(4.2) Word fluency

(5) Session E: let's get physical *(Leuven)*	(5.1) Ballgames
	(5.2) Stimulus-response

(6) Session F: quiz me quick *(Leuven)*	(6.1) Quest with riddles
	(6.2) Quiz

(7) Session G: food *(Ghent)*	(7.1) List learning
	(7.2) Order of daily activities
	(7.3) Hidden word

(8) Session H: don't worry, be happy *(Ghent)*	(8.1) Story telling
	(8.2) Awareness

(9) Session I: nature *(Ghent)*	(9.1) I spy
	(9.2) Obstacle walk
	(9.3) Opinions

(10) Session J: fun and jokes *(Ghent)*	(10.1) Buzz it
	(10.2) Problem solving
	(10.3) Choreography

(11) Session K: travel and culture *(Ghent)*	(11.1) Quest with environmental clues
	(11.2) Remember the route
	(11.3) A new language

*Note*. In italic is indicated which walking group evaluated the session.

**Table 3:** Coding tree walk-along interviews (Phase I).

Code	Subcode
Reasons for a positive experience	Difficulty was just right
	Good weather conditions
	Task promotes self-efficacy through enactive mastery

Reasons for a negative experience	Difficulty
	Duration
	Competition
	Shame/fear
	Safety
	Task hindered by…
	Childish
	Boring/monotonous
	Do not see the benefit of it/task is not perceived as mandatory/walking on its own is enough
	Weather conditions were bad

Task is not appropriate for combination with walking/would rather not do the task while walking	Because one cannot enjoy rest/silence/nature/relaxation
	Because one cannot talk enough with others
	Because one is not focused enough for performing the task
	Without specified reason

Suggestions for or comments on the cognitive tasks	Good weather conditions
	Location of the walk is important
	Social aspect is important
	The walk can take longer than one hour

**Table 4:** Coding tree focus groups (Phase II).

Theme	Subtheme
Experience of content of the cognitive tasks	Difficulty
	Feasibility
	Suggestions for improvement
	*Expectations*
	*Competition*

Experience of the cognitive walks (2-3 cognitive tasks combined in one session)	Difficulty
	Feasibility
	Suggestions for improvement
	*Expectations*
	*Social interaction*

Guidance of the cognitive walks	Instructions
	Framing of the cognitive tasks
	Coaching style

Characteristics of the program	Frequency
	Duration of the program
	Duration of one cognitive walk

Organization of the program	Communication
	Safety
	Location
	Weather conditions

Composition of the walking groups	*Group size*
	*Composition*

*Note*. Codes in italic are inductive codes.

**Table 5:** Sociodemographic information of participants in Phase II (*N* = 19).

Variable	*N* (%) unless otherwise stated
Age (years), mean (range)	73.63 (65–88)
Age groups	
65–74	11 (57.9)
75–84	7 (36.8)
85+	1 (5.3)
Region	
Ghent	10 (52.6)
Leuven	9 (47.4)
Gender	
Female	12 (63.2)
Male	7 (36.8)
Country of birth	
Belgium	18 (94.7)
France	1 (5.3)
Educational degree	
Higher secondary education (i.e., with final degree)	2 (10.5)
Postsecondary education (i.e., higher vocational education)	1 (5.3)
Higher education (i.e., university or college)	16 (84.2)
Marital status	
No relationship (i.e., single, widow(er), separated)	8 (42.1)
Relationship (i.e., married, unmarried)	11 (57.9)
Professional activity	
Professionally active	1 (5.3)
Retired	18 (94.7)

**Table 6:** Median and range of feasibility scores for all cognitive tasks rated in Phase I (*n* = 163).

Cognitive task	Feasibility	
	Median	Range
Problem solving	Median = 2.0	1.0–4.0

Immediate recall	Median = 3.0	0.0–4.0
Obstacle walk		0.0–4.0
Quest with riddles		0.0–4.0
Quiz		0.0–4.0
A new language		1.0–3.0
Awareness		1.0–4.0
Planning the route		1.0–4.0
List learning		1.0–4.0
Memory techniques		1.0–4.0
Music		1.0–4.0
Order of daily activities		1.0–4.0
Remembering the route		1.0–4.0
Spotted		2.0–4.0
Stimulus-response		2.0–4.0
Word fluency		2.0–4.0
The alphabet		3.0–4.0

Mental arithmetic	Median = 3.5	1.0–4.0
I spy		2.0–4.0

Geocaching	Median = 4.0	1.0–4.0
Serial subtraction task		1.0–4.0
Story telling		1.0–4.0
Ballgames		2.0–4.0
Choreography		2.0–4.0
Facts and titbits		2.0–4.0
N-back		2.0–4.0
Opinions		2.0–4.0
Buzz it		3.0–4.0
Hidden word		3.0–4.0
Noticing and remembering symbols		3.0–4.0
Quest with environmental clues		3.0–4.0
Word association		3.0–4.0

**Table 7:** Median and range of enjoyment scores for all cognitive tasks rated in Phase I (*n* = 163).

Cognitive task	Enjoyment	
	Median	Range
Serial subtraction task	Median = 3.0	0.0–4.0
Stimulus-response		0.0–4.0
Immediate recall		0.0–4.0
Choreography		0.0–4.0
Geocaching		1.0–4.0
N-back		1.0–4.0
Ballgames		1.0–4.0
Problem solving		1.0–4.0
Remembering the route		1.0–4.0
Mental arithmetic		1.0–4.0
List learning		1.0–4.0
Awareness		1.0–4.0
A new language		2.0–4.0
Quest with ques		2.0–4.0
Order of daily activities		2.0–4.0
Music		2.0–4.0
Spotted		2.0–4.0
Facts and titbits		3.0–4.0
I spy		3.0–4.0

Quest with riddles	Median = 3.5	0.0–4.0
Memory techniques		1.0–4.0
Opinions		2.0–4.0

Planning the route	Median = 4.0	0.0–4.0
Obstacle walk		0.0–4.0
Quiz		0.0–4.0
Story telling		1.0–4.0
The alphabet		2.0–4.0
Hidden word		2.0–4.0
Noticing and remembering symbols		2.0–4.0
Word associations		2.0–4.0
Buzz it		3.0–4.0
Word fluency		3.0–4.0

**Table 8:** Frequencies and descriptive statistics of the ratings for the walking sessions (Phase II).

Cognitive walking session	*N*	Rating of the cognitive walking session in general											Median (range)
		Percentage (%)											
		0	1	2	3	4	5	6	7	8	9	10	
Session A	17	0	0	0	0	0	12	6	**24**	18	18	**24**	8.0 (5–10)
Session B	9	0	0	0	0	0	0	0	0	22	33	**44**	9.0 (8–10)
Session C	9	0	0	0	0	0	0	0	11	11	22	**56**	10.0 (7–10)
Session D	8	0	0	0	0	0	13	13	13	13	13	**38**	8.5 (5–10)
Session E	8	0	0	0	0	0	13	0	25	25	**38**	0	8.0 (5–10)
Session F	8	0	0	0	0	0	0	13	0	13	**38**	**38**	9.0 (6–10)
Session G	7	0	0	0	0	0	0	0	0	29	0	**71**	10.0 (8–10)
Session H	7	0	0	0	0	0	0	0	14	14	29	**43**	9.0 (6–10)
Session I	9	0	0	0	0	0	11	22	**33**	0	0	**33**	7.0 (5–10)
Session J	8	0	0	0	0	0	13	0	**25**	13	**25**	**25**	8.5 (5–10)
Session K	6	0	0	0	0	0	0	0	33	0	17	50	9.5 (7–10)

*Note*. *N* = the number of persons that evaluated the cognitive walking session; the numbers in bold are the score(s) with highest percentage.

**Table 9:** Median and range of scores for all cognitive tasks on different rating scales evaluated in Phase II (*n* = 19).

Cognitive task	Difficulty		Enjoyability		Feasibility		Meaningfulness		Challenge		Interaction		Competition		Combination walking		Age appropriateness		Positive influence on the brain		Clarity of instructions	
	M	R	M	R	M	R	M	R	M	R	M	R	M	R	M	R	M	R	M	R	M	R
Music	3	1–9	10	6–10	9	6–10	9	5–10	9	2–10	9	7–10	6	1–9	9	6–10	9	8–10	8	5–10	8	6–10
Spotted	3.5	0–9	9	5–10	9	5–10	8	5–10	7	5–10	8	5–10	7.5	1–10	8	5–10	9	5–10	8.5	4–10	8.5	2–10
Plan the route	4	1–9	9	3–10	9	8–10	9	6–10	8.5	1–10	8	6–10	7	1–8	8	5–10	8.5	6–10	8.5	1–10	8	5–9
Quiz	4	0–10	9.5	7–10	9	8–10	9.5	8–10	9.5	7–10	9	0–10	8	0–9	8.5	7–10	9	8–10	9	8–10	9	8–10
Facts and titbits	5	0–9	8	5–10	9	6–10	9	5–10	8	6–10	8	4–10	6	0–8	8	4–9	9	4–10	9	4–10	9	3–10
Notice and remember symbols	5	0–9	8	5–10	9	7–10	9	7–10	8	5–10	9	5–10	7	0–10	8	7–10	8	7–10	8	7–10	9	9-10
Word associations	5	1–9	9	7–10	9	8–10	8	7–10	8	6–10	9	3–10	6	1–8	9	6–10	9	8–10	8	6–10	8	6–10
I spy	5	2–7	7	5–10	8	5–10	7	5–10	6	3–10	7	5–10	5	2–10	8	5–10	6	3–10	5	3–10	9	5–10
Mental arithmetic	5.5	1–10	7.5	6–10	8.5	7–10	7.5	6–10	7.5	5–9	7.5	3–8	7	1–9	7.5	2–10	9	7–10	8.5	5–10	8.5	6–10
Memory techniques	6	1–9	9	1–10	9	5–10	9	1–10	9	6–10	9	0–10	7	0–9	8	6–10	8	6–10	9	6–10	9	6–9
Word fluency	6	1–10	8	7–9	9	8–10	8	7–10	7	5–10	8	7–10	7	1–9	8	7–10	9	8–10	9	8–10	8	7–9
Immediate recall	6	0–10	9	7–10	10	6–10	10	7–10	9	5–10	5	0–10	5	0–10	9	7–10	8	7–10	9	7–10	10	7–10
Obstacle walk	6	3–10	9	7–10	10	7–10	9	6–10	7	5–10	7	2–10	7	2–10	7	6–10	8	5–10	8	5–10	9	7–10
Buzz it + problem solving	7	1–10	9	7–10	9	5–10	9	6–10	8	6–9	8	5–10	8	2–10	7	5–10	9	6–10	9	7–10	9	7–10
Choreography	7	2–10	9	7–10	9	7–10	9	7–10	8.5	7–10	9	4–10	8	4–10	9	7–10	9	7–10	9.5	6–10	9	7–10
A new language	7	0–10	8	6–10	9	7–10	9	6–10	8	6–10	9	6–10	7	2–10	9	7–10	9	5–10	9	6–10	8	6–10
Stimulus-response	7	1–10	9	7–10	9	8–10	8.5	5–10	8.5	1–10	7.5	5–10	7.5	1–10	9	6–10	9	7–10	9	8–10	9	8–10
Quest with riddles	7	0–10	9	7–10	9	7–10	9	7–10	9	7–10	9	7–10	7.5	0–9	9	6–10	9	7–10	9	7–10	8.5	6–10
Hidden word	7	1–10	8.5	5–10	8.5	4–10	9.5	2–10	9	2–10	9	5–10	8.5	1–10	7	5–10	8.5	5–10	9	5–10	9.5	5–10
Story telling	7	5–10	9.5	8–10	9.5	8–10	9.5	7–10	9.5	8–10	9	6–10	6	5–10	8	7–10	8	7–10	8.5	7–10	8.5	7–10
Awareness	7	5–10	8	6–10	8	7–10	8	7–10	8	6–10	8	3–10	7	3–10	8	7–10	8	7–10	8	3–10	8.5	7–10
Opinions	7	2–10	9	6–10	9	6–10	9	7–10	9	6–10	9	7–10	8	2–10	8	4–10	9	7–10	9	7–10	9	7–10
Ball-games	7.5	3–10	9	3–10	9	7–10	9	8–10	9	2–10	8.5	7–10	7.5	0–9	8	4–10	8.5	8–10	8.5	1–10	8	0–9
List learning	8	2–10	9	7–10	9	7–10	9	6–10	9	5–10	9	7–10	7	1–10	9	6–10	9	7–10	9	8–10	9	8–10
Order of daily activities	8	1–10	10	5–10	10	5–10	9	5–10	9	4–10	6.5	0–10	7.5	2–10	9	5–10	10	5–10	10	5–10	10	5–10
Quest with clues + remember the route	9	7–10	9	7–10	9	7–10	9	7–10	10	7–10	10	6–10	9.5	3–10	10	7–10	10	7–10	10	7–10	9	7–10

*Note*. M = median; R = range.

## Data Availability

The data used to support the findings of this study are available from the corresponding author upon request.

## Supplementary Materials

Supplementary Materials Supplementary File 1: example of a description of a cognitive task (Facts and titbits). Supplementary File 2: example of a summary card for a cognitively enriched walking session (Getting to know each other). Supplementary File 3: mode of enjoyability and feasibility ratings of cognitive tasks (Phase I). Supplementary File 4: mode of rating scales of cognitive tasks (Phase II). Supplementary File 5: mode of ratings of cognitively enriched walking sessions (Phase II).
