# Supplementary material for: A Two-Phased Pilot Study Evaluating the Feasibility and Acceptability of the Cognitively Enriched Walking Program “Take a Walk with Your Brain” for Older Adults
Source: J Aging Res. 2024 Apr 10;2024:2438067. doi: 10.1155/2024/2438067 (PMC11023734; doi:10.1155/2024/2438067)
Supplement: Supplementary Materials — Supplementary File 1: example of a description of a cognitive task (Facts and titbits). Supplementary File 2: example of a summary card for a cognitively enriched walking session (Getting to know each other). Supplementary File 3: mode of enjoyability and feasibility ratings of cognitive tasks (Phase I). Supplementary File 4: mode of rating scales of cognitive tasks (Phase II). Supplementary File 5: mode of ratings of cognitively enriched walking sessions (Phase II). [file 2438067.f1.pdf]

Supplementary File 1. Example of a description of a cognitive task (Facts and titbits)

This description of a cognitive task is part of the descriptions that coaches in the pilot study received in order to prepare the cognitively enriched walking sessions.

<table>
<tr><th colspan="2">FACTS AND TITBITS</th></tr>
<tr><td>Explanation</td><td>
– We start with an icebreaker: the participants are asked to share five facts and titbits about cognition and dementia with each other in pairs (+/- 2 minutes). These are available on additional cards (Appendix B).
– Next, the participants may tell one fact or titbit about themselves. These may be regular "ordinary" facts, but encourage them to think about something playful or special. Examples: date of birth, star sign, pet name, profession, favourite food, favourite holiday, allergy, phobia, morning or evening person, special talents, bizarre traits...
– The walkers are first given a short time (+/- 2 minutes) to think about which fact or titbit they want to share with each other. Then they share the fact or titbit with the other walker (still in the same pair as the facts and trivia about dementia). The other participant tries to remember this information as well as possible.
– Next, one moves on and shares the same fact or titbit with a new walking partner. Changing partners is done according to a carousel system, so that at the end everyone has heard the fact or titbit from all participants.
– At the very end of the walk, the game of "Guess who" is played (standing still with the whole group at the end of the route).
o The walkers may stand in pairs. Each pair may take turns citing a fact or titbit they have remembered from the previous walking session.
o It is up to the rest of the pairs to guess who this fact or titbit came from; the participants may discuss this in pairs.
o Which pair can correctly link all +/-5 facts or titbits to the correct participant? One must also be able to say the name of the participant! Of course, the participant from whom the fact or titbit came, may not say anything.
o This game is a fun way to end the walk, and a score can be kept as an extra competition element. Per correctly linked fact or titbit, they get a point. Which group has remembered the most?
</td></tr>
<tr><td>Instructions</td><td>
– Give the instructions after walking for some minutes.
– Instructions:
</td></tr>
</table>

	o "During this task, we will get to know each other a little better. You get to walk in pairs. Together, you briefly discover some facts and trivia about dementia and cognition. Then you tell the other one fact or titbit about yourself. Try to remember the facts or titbits about the other, because at the end we will do a little quiz! After this, you may move on to the next participant. You tell them the same fact about yourself as before, and listen to the fact about the other participant."<br>o "As mentioned earlier, we will now play "Guess who". Stand in pairs, and together choose a fact that you have memorized about someone else. Each pair may take turns sharing that fact with the group, the other pairs must guess who that fact or titbit was about. For this, they may deliberate briefly."<br>– Give the following framing for the cognitive task:<br>o "In this task, participants are encouraged to really listen to each other and try to learn and remember as much as possible from each other. Not only will this lead to better acquaintance, it is also very good for the brain. This is because you use your autobiographical memory; this memory contains memories of specific events or trivia you have learned in the course of your own life. In addition, short-term memory is called upon as you try to remember new facts and titbits introduced by other participants. Several experts therefore recommended this exercise to keep the brain active."<br>– Start the task. Mind the timing, make sure you give the needed guidance and mind the important considerations listed below.
Duration	– Icebreaker (facts and titbits about dementia and cognition): 2 minutes<br>– Facts and titbits about themselves: 13 minutes (1 minute per pair + 9x change so everyone had the chance to share their fact with every participant)<br>– Endgame "Guess who": 5 minutes
Variation	– In case it is too easy in pairs, you can also let everyone play the game "Guess who" individually<br>– You can make it more difficult by increasing the amount of facts/titbits to remember, and make it more easy by decreasing the amount of facts/titbits to remember

Material needed	– Additional cards with facts and titbits about dementia and cognition (5x)

	– Smartphone or paper to keep the score and to write down the facts/titbits	
Preparation	– You can provide additional questions to help participants that don't know which fact or titbit they can tell (for inspiration, see the examples above).	
Guidance	– After explaining the task, the coach has to provide assistance in case participants don't know which fact or titbit they can tell. The coach can also share a fact or titbit about him/herself. He/she also writes down the facts/titbits of the participants as a check for the "Guess who" game.	
Important considerations	GENERAL<br>– Make sure everyone can share a fact or titbit and that the participants listen to each other<br>– Make sure that the facts or titbits are not too extensive and that everyone shares the same fact or titbit with every participant.	INDIVIDUAL<br>– Every participant has to share a fact or titbit.<br>– You can help participants that aren't sure about which fact or titbit to share by asking additional quesions.

Supplementary File 2. Example of a summary card for a cognitively enriched walking session (Session 1 - Getting to know each other)

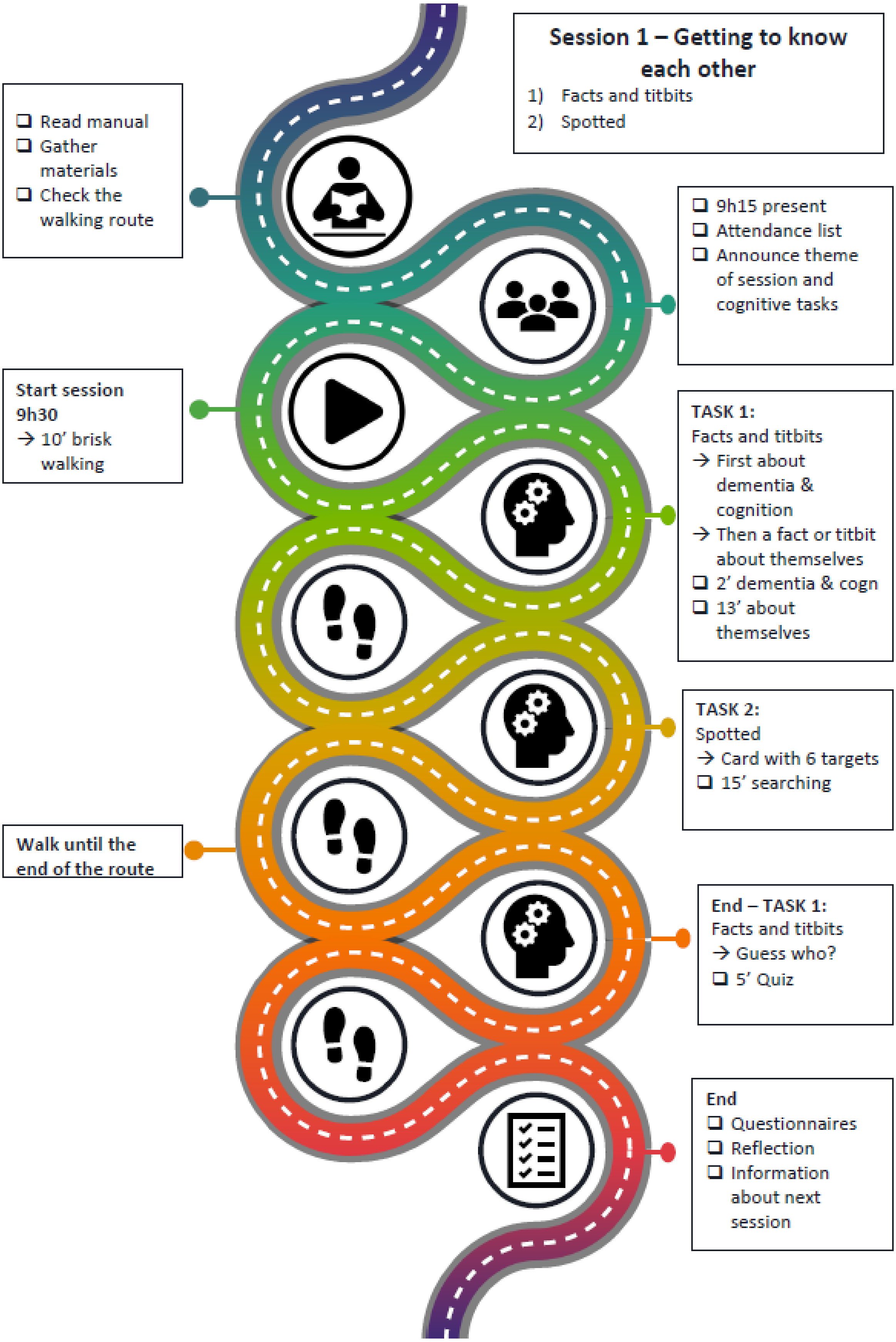

Supplementary File 3. Mode of enjoyability and feasibility ratings of cognitive tasks Phase I

Cognitive task	Enjoyability Mode	Feasibility Mode
A new language	3.00	3.00
Awareness	4.00	3.00
Ball-games	4.00	4.00
Buzz it	4.00	4.00
Choreography	3.00	4.00
Facts and titbits	3.00	4.00
Geocaching	3.00*	4.00
Hidden word	4.00	4.00
I spy	3.00	4.00
Immediate recall	3.00	3.00
List learning	3.00	3.00
Memory techniques	4.00	3.00*
Mental arithmetic	4.00	4.00
Music	3.00*	3.00
N-back	3.00	4.00
Noticing and remembering symbols	4.00	4.00
Obstacle walk	4.00	4.00
Opinions	4.00	4.00
Order of daily activities	3.00	3.00*
Planning the route	4.00	3.00*
Quest with clues	4.00	4.00
Quest with riddles	4.00	3.00
Quiz	4.00	3.00*
Remembering the route	3.00*	3.00*
Serial subtraction task	3.00	4.00
Solving riddles	3.00	2.00
Spotted	3.00	3.00
Stimulus-response	3.00	3.00*
Story telling	4.00	4.00
The alphabet	4.00	3.00
Word fluency	4.00	3.00*
Wordassociations	4.00	4.00

*Multiple modes exist, the smallest value is shown

Supplementary File 4. Mode of rating scales of cognitive tasks Phase II

	**Phase II Rating scales, Mode**										
**Cognitive task**	**Enjoy-ability**	**Feasibility**	**Meaning-fulness**	**Challenge**	**Difficulty**	**Interaction**	**Compe-tition**	**Combi-nation walking**	**Age appro-priateness**	**Positive influence on brain**	**Clarity of instructions**
Facts and titbits	8	9	9	6*	0*	8	8	8	9	9	9
Spotted	9	9	8	7*	1	9	8*	8	9	10	8
List learning	9	9	9	9	8	7*	7	10	9	9	9
Notice and remember symbols	8	10	10	8	1*	9	9	8	8	8	9
Memory techniques	9	9	9	9	1*	9	7	8*	7	9	9
Mental arithmetic	7	8	7	6*	1	8	9	6*	7*	9	6*
Music	10	9*	9	9	7	9	9	9	9	8	8
Word associations	10	10	8	6*	1*	9	6	9	9	8	8
Plan the route	10	9	9	9	1	8	8	8	8*	9	9
Word fluency	9	9	7*	7	1*	7*	7*	8	8*	9	8*
Ball-games	9	9	8*	9	10	8	8	9	8	9	8
Stimulus-response	9	9	8*	9*	7*	7*	1*	9*	9	9	9
Quest with riddles	9	9	9	9*	7	9	9	9	9	9*	8*
Quiz	10	8*	10	10	1	9	8	8	9	9	9
Immediate recall	10	10	10	9	0*	0*	5	10	8	8*	10
Order of daily activities	10	10	10	10	9*	0*	5*	10	10	10	10
Hidden word	5*	10	10	9*	10	5*	10	5	10	10	10
Story telling	10	10	10	10	6*	10	5*	7*	7*	8*	8*
Awareness	6*	7	7*	10	10	10	3	8*	8*	10	8*
I spy	5*	10	5	10	2*	7*	5*	10	10	10	10

Cognitive task	Enjoy-ability	Feasibility	Meaning-fulness	Challenge	Difficulty	Interaction	Compe-tition	Combi-nation walking	Age appro-priateness	Positive influence on brain	Clarity of instructions
Obstacle walk	10	10	10	6*	5*	5*	5*	7	10	10	10
Opinions	10	9*	10	10	7*	10	8*	9	9*	10	9*
Buzz it + problem solving	7	10	9	9	7	8*	5*	7	9	9	10
Choreography	9	9	9	7	7*	9*	4*	9	10	10	9*
Quest with clues + remember the route	9	9	10	10	7	10	10	10	10	10	7*
A new language	7*	7*	7*	6*	7	10	6*	7*	10	10	8*

*Multiple modes exist, the smallest value is shown

Supplementary File 5. Mode of ratings of cognitively enriched walking sessions Phase II

Cognitive walking session	Mode
**Session A**	7*
**Session B**	10
**Session C**	10
**Session D**	10
**Session E**	9
**Session F**	9*
**Session G**	10
**Session H**	10
**Session I**	7
**Session J**	7
**Session K**	10

*Multiple modes exist, the smallest value is shown
